# Heat Stress Induces Metabolic and Physiological Imbalance in Laying Hens, Accompanied by Hepatic Transcriptomic, Cecal Microbial, and Metabolomic Alterations

**DOI:** 10.3390/ani16111578

**Published:** 2026-05-22

**Authors:** Zi Mei, Haobo Zhou, Hao Du, Kunyuan Liu, Chaoyang Gao, Zheya Sheng, Yanzhang Gong

**Affiliations:** Key Laboratory of Agricultural Animal Genetics, Breeding and Reproduction, Ministry of Education, College of Animal Science and Technology and College of Veterinary Medicine, Huazhong Agricultural University, Wuhan 430070, China

**Keywords:** heat stress, hens, liver, intestinal microbiota, metabolism

## Abstract

Heat stress is a major constraint to productivity and physiological homeostasis in laying hens. This study investigated integrated responses to acute heat stress using a multi-omics approach, including performance traits, serum biochemical parameters, histology, hepatic transcriptomics, cecal metagenomics, and metabolomics. Acute heat stress impaired productive performance, as reflected by changes in egg production and reduced eggshell strength, and induced systemic physiological disturbances, including increased stress- and injury-related blood indicators and disrupted metabolic and electrolyte balance. Histological analysis confirmed liver and intestinal tissue damage. Hepatic transcriptomics revealed inflammatory activation and suppression of metabolic pathways, particularly those involved in lipid metabolism, energy production, and redox homeostasis. Cecal metagenomic and metabolomic analyses showed altered microbial composition and functional potential, along with disruptions in amino acid, lipid, and energy metabolism. Collectively, these findings suggest that acute heat stress is associated with coordinated inflammatory responses and metabolic reprogramming, together with liver and intestinal injury and gut microbiota–metabolite alterations. The study provides a framework for understanding early heat stress responses and highlights potential targets for nutritional and microbiota-based interventions in poultry production. Importantly, serum biochemical indicators such as D-lactic acid and aspartate aminotransferase may serve as potential early biomarkers for monitoring heat-stress-induced physiological disturbances.

## Simple Summary

This study evaluated the multi-level effects of acute heat stress on laying hens. Heat stress reduced laying performance and altered stress-related blood biochemical indicators. It also induced tissue damage, as reflected by increased serum injury markers (AST, D-lactic acid, and diamine oxidase), along with histological changes in the liver and intestine. In addition, acute heat stress altered cecal microbial composition and affected energy-related metabolites. Liver transcriptomic analysis further showed changes in immune-related and metabolic pathways, with upregulation of genes involved in inflammatory responses and downregulation of genes associated with lipid metabolism and energy production. Overall, these findings indicate that acute heat stress disrupts physiological, structural, and metabolic homeostasis in laying hens.

## 1. Introduction

Heat stress is increasingly recognized as one of the major environmental constraints on modern poultry production [1,2,3,4,5,6]. Because laying hens maintain a high metabolic rate to support continuous egg formation, exposure to temperatures above the thermoneutral zone can reduce feed intake, egg production, and egg quality, while also compromising welfare and health [7,8,9]. As hot weather events become more frequent and prolonged, heat stress has become a persistent threat to the sustainability of egg production systems [10,11].

Beyond its negative effects on productive performance, heat stress disrupts systemic homeostasis through endocrine, oxidative, and metabolic responses. Increased corticosterone secretion, excessive reactive oxygen species production, and disturbances in acid–base and electrolyte balance have all been associated with reduced physiological resilience in poultry [12,13]. The gastrointestinal tract is particularly vulnerable to thermal challenge because hyperthermia and blood flow redistribution can damage villus structure, loosen tight junctions, and increase intestinal permeability [14,15]. Such changes may impair nutrient absorption and facilitate the translocation of luminal antigens and microbial products, thereby amplifying inflammatory responses [16,17,18].

The liver is another important target organ during heat stress [19,20,21]. As the central hub of lipid, amino acid, antioxidant, and energy metabolism in birds, the liver plays a critical role in maintaining whole-body metabolic homeostasis [22,23,24,25]. Previous transcriptomic and integrative omics studies in chickens have shown that heat stress reshapes hepatic pathways related to immune regulation, oxidative balance, and nutrient metabolism [26,27,28]. However, most available evidence has been derived from broilers or from studies focusing on a single tissue, and the hepatic molecular response to heat stress in laying hens remains less well characterized.

At the same time, accumulating evidence indicates that heat stress perturbs the intestinal microbial ecosystem [29,30]. In laying hens, both cecal and fecal microbial profiles, as well as microbial functional pathways, have been reported to shift under high-temperature exposure [31]. Notably, one study suggested that part of the cecal microbial response in laying hens may be driven by heat-stress-associated feed restriction rather than by thermal load alone, whereas other studies support a more direct contribution of heat stress to intestinal injury and microbiota-associated dysfunction [32]. This unresolved issue suggests that microbial alterations should be interpreted together with host phenotypes and downstream metabolic outputs.

Multi-omics strategies are increasingly being applied to dissect the complex biological consequences of heat stress in poultry. Integrated microbiota–transcriptome–metabolome analyses have shown that heat exposure can simultaneously alter microbial communities, host gene expression, and metabolic pathways [17,33]. Nevertheless, comparable integrative evidence in laying hens—particularly studies that jointly evaluate productive performance, tissue injury, hepatic transcriptomic responses, cecal microbial function, and cecal metabolic alterations—remains limited.

Therefore, the present study investigated the effects of heat stress on laying hens using a combined phenotypic, histological, hepatic transcriptomic, cecal metagenomic, and cecal metabolomic approach. We hypothesized that heat stress would impair laying performance, induce liver and intestinal injury, activate inflammatory signaling while suppressing metabolic pathways in the liver, and be accompanied by marked shifts in cecal microbial composition, microbial functional potential, and metabolite profiles.

## 2. Materials and Methods

### 2.1. Animals and Experimental Design

Seventy 28-week-old Xinhua No. 2 laying hens (a local commercial strain with a maturing age of approximately 151 days) were obtained from Hubei Xinhua Ecological Animal Husbandry Development Co., Ltd., Jingmen, China. All birds were acclimated for 2 weeks under thermoneutral conditions (23 ± 1 °C, 60–70% relative humidity, 15 h light/9 h dark) before the experiment. The trial was conducted in a climate-controlled chamber at the experimental poultry farm of Huazhong Agricultural University. The hens were randomly divided into two groups: a control group (CON, *n* = 35) and a heat stress group (HS, *n* = 35). All hens were housed in wire cages (70 cm × 70 cm × 30 cm) with two birds per cage, providing a stocking density of 2450 cm^2^/bird. According to our previous model, acute heat stress was induced by exposing hens to 36 °C for 6 h under a relative humidity of approximately 70%. The control (CON) group was maintained at 23 ± 1 °C under the same humidity conditions. Feed and water were available ad libitum throughout the experiment.

### 2.2. Sample Collection

At the end of the 6 h heat stress exposure, rectal temperature was measured in all hens (*n* = 35 per group). Blood samples were collected from the wing vein of all hens for blood gas and serum biochemical analyses. Whole blood for blood gas analysis was collected into lithium heparin tubes, whereas blood for serum biochemical analysis was collected into plain tubes [34]. Serum was obtained after centrifugation for subsequent assays. In addition, four hens per group (*n* = 4) were randomly selected and euthanized by exsanguination under anesthesia [12]. Liver tissue and intestinal segments, including the duodenum, jejunum, and ileum, were collected immediately for further analyses [12].

### 2.3. Measurement of Blood Gas Parameters in Anticoagulated Whole Blood

Whole blood collected in lithium heparin tubes was gently inverted several times to ensure complete mixing. Then, 90 μL of whole blood was aspirated using a micropipette and loaded into the sample well of a CG8+ blood gas test strip. The strip was immediately inserted into an i-STAT 1 portable blood analyzer (Abbott, Chicago, IL, USA), and the blood gas parameters were automatically measured and recorded [34].

### 2.4. Serum Biochemical Analysis

For serum biochemical analysis, blood collected in plain tubes was allowed to clot and then centrifuged at 3000 rpm for 10 min to obtain serum. Subsequently, 500 μL of each serum sample was transferred into a dedicated biochemical sample cup and analyzed using an automatic biochemical analyzer (BX-4000, Sysmex, Kobe, Japan) [34]. Commercial reagent kits (Huaxin Dibao Biotechnology Co., Ltd., Guangzhou, China) were used to determine the concentrations or activities of aspartate aminotransferase (AST, DE0171, Huaxin Dibao Biotechnology Co., Ltd., Guangzhou, China), total cholesterol (TCHO, DN014, Huaxin Dibao Biotechnology Co., Ltd.), triglycerides (TG, DN012, Huaxin Dibao Biotechnology Co., Ltd.), glucose (GLU, DN022, Huaxin Dibao Biotechnology Co., Ltd.), creatine kinase (CK, DN032, Huaxin Dibao Biotechnology Co., Ltd.), and lactate dehydrogenase (LDH, DN044, Huaxin Dibao Biotechnology Co., Ltd.), according to the manufacturer’s instructions. The intra- and inter-assay coefficients of variation (CVs) were <5% and <10%, respectively, with an analytical sensitivity of 0.015 ΔA/min and accuracy within 10% relative deviation from calibration standards.

### 2.5. Determination of Serum CORT, DAO, DLA, CAT, and SOD

Serum concentrations or activities of corticosterone (CORT), diamine oxidase (DAO), D-lactic acid (DLA), catalase (CAT), and superoxide dismutase (SOD) were determined using commercially available assay kits according to the manufacturers’ instructions. Specifically, DAO (F4389), CORT (F4367), and DLA (F4395) kits were purchased from Shanghai Fankewei Biotechnology Co., Ltd. (Shanghai, China), while CAT (S0051) and SOD (S0086) kits were obtained from Beyotime Biotechnology (Shanghai, China). Absorbance was measured using a microplate reader (Spark 350, Tecan, Männedorf, Switzerland), and concentrations or activities were calculated based on the corresponding standard curves. The intra- and inter-assay CVs were both <15%, and all measurements were performed in duplicate.

### 2.6. Measurement of Laying Performance and Egg Quality

Eggs from the CON and HS groups were collected during the 24 h period immediately following the 6 h acute heat stress exposure. This period was defined as the post-stress response window. The laying rate was calculated as the number of eggs produced during this 24 h period divided by the total number of hens in each group (*n* = 35). No pre-exposure or baseline laying performance was included in this calculation. Egg weight was measured using an electronic balance. Eggshell strength was determined using an eggshell strength tester (EFG0503, Bulader Technology Development Co., Ltd., Beijing, China) according to the manufacturer’s instructions [35].

### 2.7. Histological Analysis

Liver tissue and intestinal segments, including the duodenum, jejunum, and ileum, were fixed in 4% paraformaldehyde for 24 h at room temperature, dehydrated in a graded ethanol series, cleared in xylene, and embedded in paraffin. Paraffin blocks were sectioned at 6 μm using a microtome (Leica RM2235, Leica Microsystems, Wetzlar, Germany) and stained with hematoxylin and eosin following standard procedures [12]. Histopathological changes in the liver and intestine were examined under a light microscope (Olympus BX53, Olympus Corporation, Tokyo, Japan). Representative images were recorded, and villus height and crypt depth of the duodenum, jejunum, and ileum were quantified using ImageJ software (National Institutes of Health, Bethesda, MD, USA, version 1.53) [12].

### 2.8. Hepatic Transcriptome Sequencing and Differential Expression Analysis

Total RNA was extracted from liver tissues (*n* = 4 per group) using TRIzol reagent (Invitrogen, Thermo Fisher Scientific, Waltham, MA, USA). RNA quality and concentration were assessed using a NanoDrop spectrophotometer, 1% agarose gel electrophoresis, and an Agilent 2100 Bioanalyzer (Agilent Technologies, Santa Clara, CA, USA). Qualified RNA samples were used for library construction with the NEBNext^®^ Ultra™ RNA Library Prep Kit for Illumina^®^ (New England Biolabs, Ipswich, MA, USA). After mRNA enrichment, fragmentation, and cDNA synthesis, libraries were sequenced on the Illumina HiSeq X Ten platform with a paired-end 150 bp strategy. Raw reads were filtered using fastp (v0.20.0) to obtain clean reads, which were aligned to the *Gallus gallus* reference genome (GRCg6a) using HISAT2 (v2.0.5) [36]. Gene expression was quantified using featureCounts (v1.5.0-p3) and expressed as fragments per kilobase of transcript per million mapped reads (FPKM) [37]. Differential expression analysis was performed using DESeq2 (v1.20.0), and genes with an adjusted *p* value < 0.05 and |log2FoldChange| ≥ 1 were identified as differentially expressed genes (DEGs) [38]. Functional enrichment analyses of DEGs, including Gene Ontology (GO) and Kyoto Encyclopedia of Genes and Genomes (KEGG) pathways, were performed using clusterProfiler (v3.8.1) [39].

### 2.9. Functional Enrichment and Gene Set Enrichment Analyses

Gene Ontology (GO) and Kyoto Encyclopedia of Genes and Genomes (KEGG) enrichment analyses were performed using the clusterProfiler package (v4.8.1). Significantly enriched GO terms and KEGG pathways among differentially expressed genes were identified using the hypergeometric test, with *p* < 0.05 considered significant.

Gene set enrichment analysis (GSEA) was also conducted using the clusterProfiler package (version 4.8.1) in R to evaluate pathway-level changes across the whole transcriptome. All genes were ranked according to their differential expression between groups, and KEGG pathway gene sets were used as the reference database. The normalized enrichment score (NES), nominal *p* value, and false discovery rate (FDR) were calculated for each pathway. Pathways with nominal *p* < 0.05 and FDR < 0.25 were considered significantly enriched.

### 2.10. RNA Reverse Transcription and Quantitative Real-Time PCR

For mRNA analysis, first-strand cDNA was synthesized from 1 μg of total RNA using the HiScript^®^ III RT SuperMix Kit (Vazyme, Nanjing, China) according to the manufacturer’s instructions. Quantitative real-time PCR was performed using a Bio-Rad CFX-384 system (Bio-Rad Laboratories, Hercules, CA, USA) with 2 × SYBR Green Fast qPCR Mix (ABclonal Biotechnology Co., Ltd., Wuhan, China). Each sample was analyzed in triplicate. The amplification protocol consisted of an initial denaturation at 95 °C for 3 min, followed by 40 cycles of 95 °C for 10 s and 60 °C for 30 s. Melting curve analysis was performed to confirm amplification specificity. Relative mRNA expression levels were calculated using the 2^−ΔΔCt^ method. β-actin was used as the internal reference gene based on previous literature in heat stress-related studies and its stable expression under the experimental conditions. [40,41]. Primer sequences, melting temperatures (Tm), and expected amplicon sizes are provided in Table A1. All primers were designed de novo based on species-specific coding sequences (CDS) retrieved from the NCBI database using Primer3Plus software (https://www.primer3plus.com/, accessed on 10 May 2025).

### 2.11. Cecal Metagenomic Sequencing and Bioinformatic Analysis

For metagenomic analysis, cecal contents were collected from hens (*n* = 4 per group) in the CON and HS groups. Microbial genomic DNA was extracted using a commercial DNA extraction kit according to the manufacturer’s instructions. DNA concentration and quality were assessed using a Qubit 4 fluorometer and agarose gel electrophoresis. Sequencing libraries with an average insert size of approximately 350 bp were constructed and sequenced on the Illumina NovaSeq platform with a paired-end 150 bp strategy. Raw reads were quality-filtered using fastp (v0.23.1) to obtain clean reads. Host-derived sequences were removed by aligning clean reads to the *Gallus gallus* reference genome (GRCg6a) using Bowtie2 (v2.2.4). High-quality reads were de novo assembled using MEGAHIT (v1.2.9), and open reading frames (ORFs) were predicted using GeneMark.hmm (v2.1) [42,43]. Redundant sequences were removed using CD-HIT (v4.5.8) to construct a non-redundant gene catalog [44]. Predicted protein sequences were aligned against the NCBI NR, KEGG, eggNOG, and CAZy databases using DIAMOND (v2.1.6) for taxonomic and functional annotations [45,46,47,48]. Taxonomic assignment was determined using the last common ancestor (LCA) algorithm. Principal component analysis (PCA) and microbial diversity analysis, including the identification of differential taxa and functional profiles, were performed using R software (v2.15.3 and v3.0.3) and LEfSe (Galaxy implementation, http://huttenhower.sph.harvard.edu/galaxy/, accessed on 18 June 2025) [49].

### 2.12. Untargeted Metabolomic Analysis of Cecal Contents

For untargeted metabolomic analysis, cecal contents were collected from hens (*n* = 4 per group) in the CON and HS groups. Approximately 20 mg of each sample was extracted with 400 μL of methanol:water (7:3, *v*/*v*) containing internal standards. Missing values were imputed using the KNN method (impute package, v1.56.0). Before multivariate analysis, metabolite abundances were normalized to internal standards, log2-transformed, and mean-centered. Principal component analysis (PCA, base package v4.1.2) and orthogonal partial least squares discriminant analysis (OPLS-DA, MetaboAnalystR v1.0.1) were performed, with data subjected to unit variance (UV) scaling to visualize overall metabolic patterns and maximize group separation [50]. Differential metabolites were screened using the criteria of variable importance in projection (VIP ≥ 1), |fold change| ≥ 2, and *p* value < 0.05 (Student’s *t*-test). Hierarchical clustering heatmaps were generated using the ComplexHeatmap package (v2.9.4). Differential metabolites were further subjected to pathway enrichment analysis using MetaboAnalyst 6.0. Correlation analyses, including Spearman’s correlations and network visualizations, were performed using the corrplot (v0.92), fmsb (v0.71), and igraph (v1.2.11) packages.

### 2.13. Statistical Analysis

Data are presented as mean ± standard deviation (SD), with each hen considered an experimental unit. Statistical analyses were performed using SPSS 26.0 (IBM Corp., Armonk, NY, USA) and GraphPad Prism 8.0.2. Differences between the CON and HS groups were analyzed using Student’s *t*-test. A *p* value < 0.05 was considered statistically significant.

## 3. Results

### 3.1. Heat Stress Altered Productive Performance and Serum Physiological Indicators in Laying Hens

**Figure 1:**
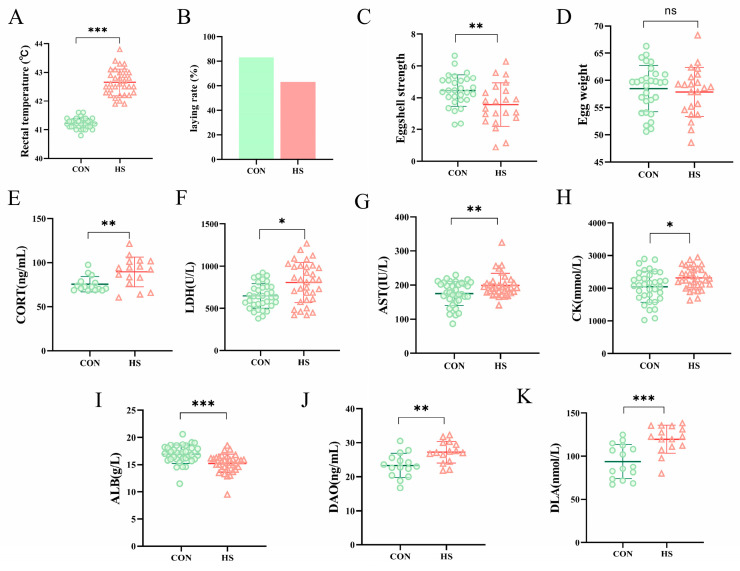
Effects of heat stress on productive performance and serum physiological indicators in laying hens. (**A**) Rectal temperature. (**B**) Laying rate. (**C**) Eggshell strength. (**D**) Egg weight. (**E**) Serum corticosterone (CORT). (**F**) Serum lactate dehydrogenase (LDH). (**G**) Serum aspartate aminotransferase (AST). (**H**) Serum creatine kinase (CK). (**I**) Serum albumin (ALB). (**J**) Serum diamine oxidase (DAO). (**K**) Serum D-lactic acid (DLA). Data are shown as mean ± SD. * *p* < 0.05, ** *p* < 0.01, *** *p* < 0.001; ns, not significant.

Heat stress significantly affected productive performance and serum physiological indicators in laying hens. Compared with the CON group, rectal temperature was significantly increased in the HS group (*p* < 0.001) (Figure 1A), confirming successful induction of acute heat stress. For productive performance, egg production during the 24 h period following heat exposure showed a numerical reduction in the HS group; however, this difference did not reach statistical significance (*p* > 0.05). In contrast, eggshell strength was significantly decreased (*p* < 0.05), whereas egg weight was not affected (Figure 1B–D). Furthermore, heat stress induced marked biochemical disturbances, as evidenced by significant increases in serum concentrations of corticosterone (CORT, *p* < 0.01), lactate dehydrogenase (LDH, *p* < 0.05), aspartate aminotransferase (AST, *p* < 0.01), creatine kinase (CK, *p* < 0.05), diamine oxidase (DAO, *p* < 0.01), and D-lactic acid (DLA, *p* < 0.001) (Figure 1E–K). In contrast, serum albumin (ALB) concentrations significantly decreased (*p* < 0.001) (Figure 1I). These results indicated that while acute heat stress primarily impacted egg quality and physiological homeostasis, it also initiated a negative trend in overall productive performance.

### 3.2. Heat Stress Altered Blood Gas, Electrolyte, and Serum Metabolic Parameters in Laying Hens

**Figure 2:**
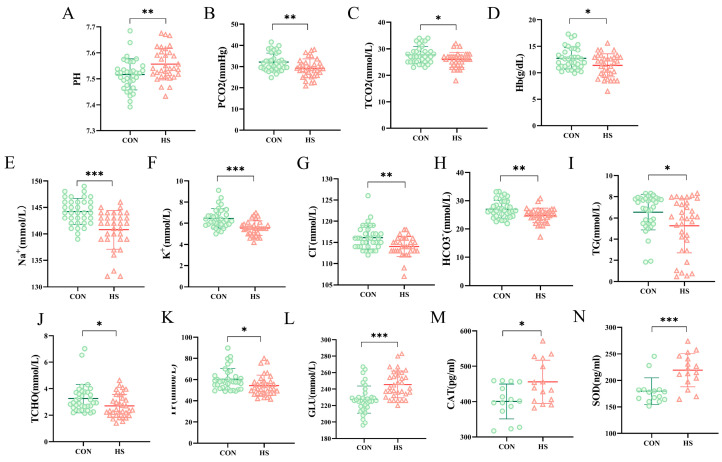
Effects of heat stress on blood gas, electrolyte, and serum metabolic parameters in laying hens. (**A**) Blood pH. (**B**) PCO2. (**C**) TCO2. (**D**) Hemoglobin (Hb). (**E**) Na^+^. (**F**) K^+^. (**G**) Cl^−^. (**H**) HCO3^−^. (**I**) Triglyceride (TG). (**J**) Total cholesterol (TCHO). (**K**) Total protein (TP). (**L**) Glucose (GLU). (**M**) Catalase (CAT). (**N**) Superoxide dismutase (SOD). Data are shown as mean ± SD. * *p* < 0.05, ** *p* < 0.01, *** *p* < 0.001.

Heat stress significantly affected blood gas, electrolyte, and serum metabolic parameters in laying hens. Compared with the CON group, the HS group showed a significantly higher blood pH (*p* < 0.01), whereas the partial pressure of carbon dioxide (PCO_2_, *p* < 0.01), total carbon dioxide (TCO_2_, *p* < 0.05), and hemoglobin (Hb, *p* < 0.05) were significantly decreased (Figure 2A–D). In addition, the concentrations of Na^+^ (*p* < 0.001), K^+^ (*p* < 0.001), Cl^−^ (*p* < 0.01), and HCO_3_^−^ (*p* < 0.01) were significantly reduced in the HS group (Figure 2E–H). Moreover, heat stress significantly decreased serum triglyceride (TG, *p* < 0.05), total cholesterol (TCHO, *p* < 0.05), and total protein (TP, *p* < 0.05) concentrations, whereas serum glucose (GLU, *p* < 0.001), catalase (CAT, *p* < 0.05), and superoxide dismutase (SOD, *p* < 0.001) were significantly increased in the HS group (Figure 2I–N).

### 3.3. Heat Stress Induced Liver Injury and Altered Intestinal Morphology

**Figure 3:**
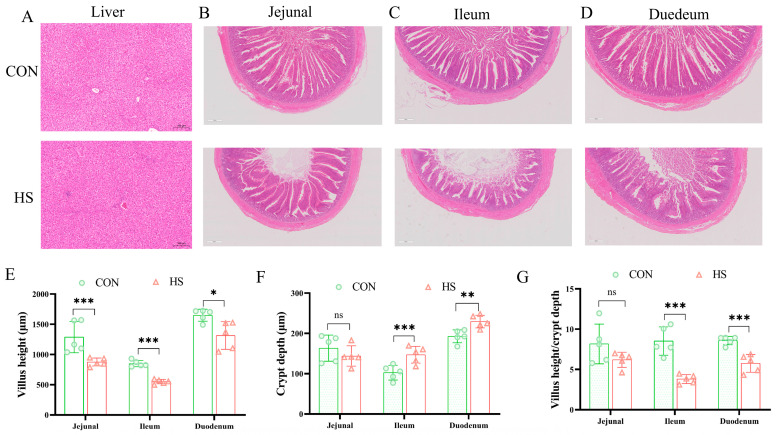
Effects of heat stress on liver histopathology and intestinal morphology in laying hens. (**A**) Representative hematoxylin and eosin stained images of the liver. (**B**) Representative H&E-stained images of the jejunum. (**C**) Representative H&E-stained images of the ileum. (**D**) Representative H&E-stained images of the duodenum. (**E**) Villus height. (**F**) Crypt depth. (**G**) Villus height/crypt depth ratio. Data are presented as mean ± SD. * *p* < 0.05, ** *p* < 0.01, *** *p* < 0.001; ns, not significant.

H&E staining revealed evident histopathological alterations in the liver following heat stress. Compared with the CON group, hepatic tissue in the HS group exhibited a small number of inflammatory foci, extensive hepatocellular steatosis, and numerous small round cytoplasmic vacuoles, indicating fatty degeneration (Figure 3A). Occasional venous congestion was also observed. Heat stress also markedly altered intestinal morphology (Figure 3B–D). Villus height was significantly reduced in the jejunum (*p* < 0.001), ileum (*p* < 0.001), and duodenum (*p* < 0.05) after heat stress (Figure 3E). Crypt depth was significantly increased in the ileum (*p* < 0.001) and duodenum (*p* < 0.01), but remained unchanged in the jejunum (Figure 3F). The villus height/crypt depth ratio was significantly decreased in the ileum and duodenum of the HS group (*p* < 0.001), whereas no significant difference was observed in the jejunum (Figure 3G).

### 3.4. Heat Stress Altered the Hepatic Transcriptomic Profile

**Figure 4:**
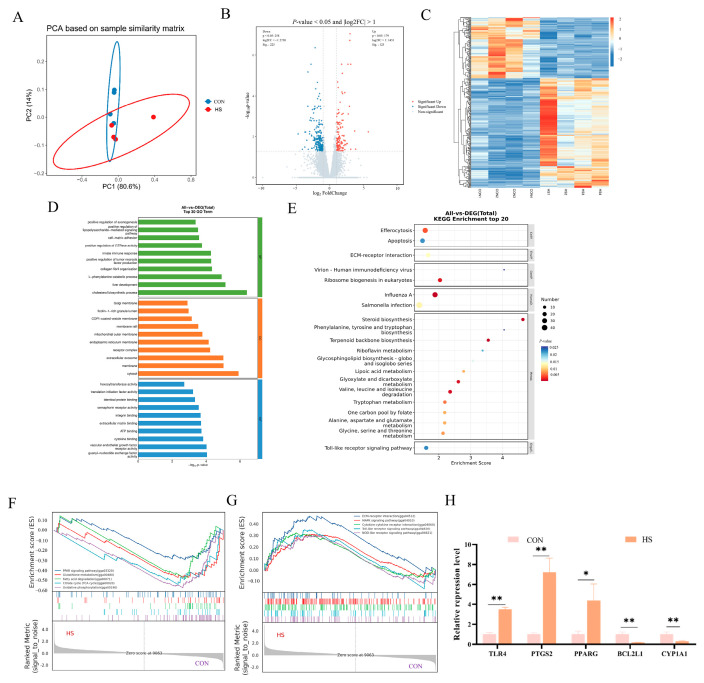
Hepatic transcriptomic landscape and validation in laying hens under acute heat stress. (**A**) Principal Component Analysis (PCA) score plot showing the clustering of samples from the control (Con) and heat stress (HS) groups. (**B**) Volcano plot of differentially expressed genes (DEGs). Red and blue dots represent significantly up-regulated and down-regulated genes. (**C**) Hierarchical clustering heatmap of DEGs showing expression patterns across individual samples. (**D**) Top 30 significantly enriched Gene Ontology (GO) terms. (**E**) Top 20 significantly enriched Kyoto Encyclopedia of Genes and Genomes (KEGG) pathways. (**F**) Gene set enrichment analysis (GSEA) of pathways suppressed by heat stress. (**G**) GSEA of pathways activated by heat stress. (**H**) qRT-PCR validation of selected genes. Data are shown as mean ± SD (*n* = 4 per group). Statistical significance was determined using Student’s *t*-test, where * *p* < 0.05 and ** *p* < 0.01 represent significant differences.

To further investigate the hepatic response to heat stress, RNA-seq analysis was performed on liver tissues from the CON and HS groups, with four biological replicates per group. After quality control, approximately 333 million clean reads were obtained in total, with 41–43 million clean reads for each sample. All samples showed high sequencing quality, with Q20 and Q30 values above 90%, and mapping rates to the *Gallus gallus* reference genome (GRCg6a) exceeding 90%, indicating that the data were suitable for subsequent analysis (Table 1). PCA and hierarchical clustering analysis revealed clear differences in hepatic gene expression patterns between the two groups (Figure 4A). Based on the criteria of Padj < 0.05 and |log2FC| > 1, a total of 350 differentially expressed genes (DEGs) were identified, including 125 upregulated and 225 downregulated genes in the HS group (Figure 4B,C). GO analysis showed that these DEGs were mainly enriched in innate immune response, positive regulation of tumor necrosis factor production, cell–matrix adhesion, collagen fibril organization, liver development, and cholesterol biosynthetic process. KEGG analysis further indicated significant enrichment in apoptosis, efferocytosis, ECM–receptor interaction, Toll-like receptor signaling, steroid biosynthesis, and several metabolism-related pathways (Figure 4D,E). Moreover, GSEA showed activation of ECM–receptor interaction, MAPK signaling, cytokine–cytokine receptor interaction, Toll-like receptor signaling, and NOD-like receptor signaling, together with inhibition of PPAR signaling, glutathione metabolism, fatty acid degradation, the citrate cycle, and oxidative phosphorylation in the HS group (Figure 4F,G). In addition, qRT-PCR validation of selected genes showed expression trends consistent with the RNA-seq results (Figure 4H).

### 3.5. Heat Stress Altered the Intestinal Microbial Community Structure and Functional Profile

**Figure 5:**
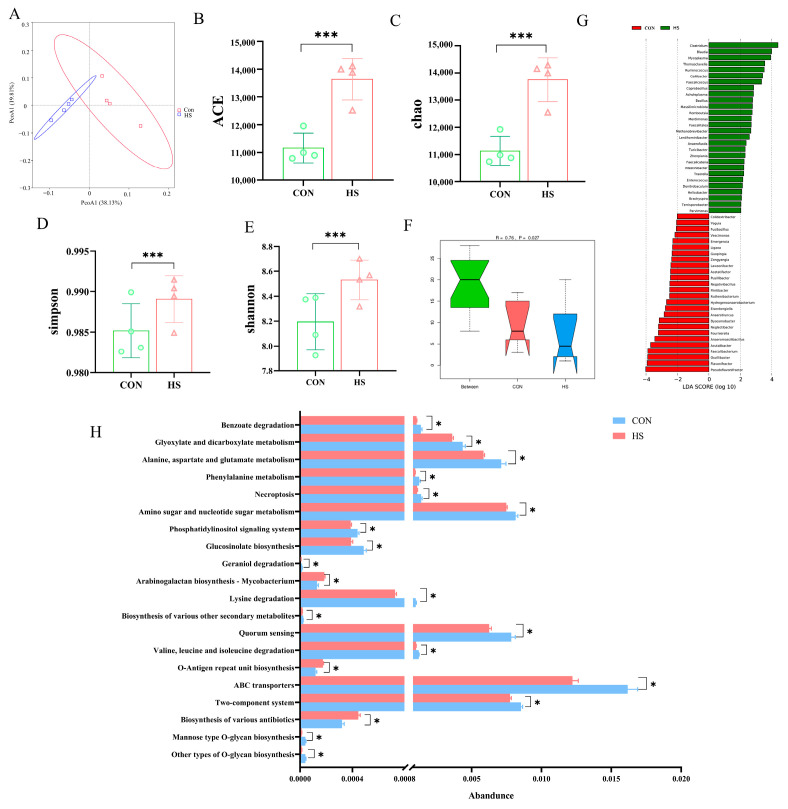
Heat stress altered the cecal microbial community structure and functional profile in laying hens. (**A**) PCoA plot of microbial beta diversity. (**B**) ACE index. (**C**) Chao index. (**D**) Simpson index. (**E**) Shannon index. (**F**) ANOSIM analysis of microbial community structure between groups. (**G**) LEfSe analysis of differential genera at the genus level. (**H**) Relative abundances of differential KEGG functional pathways in the cecal microbiota. * *p* < 0.05, *** *p* < 0.001.

To investigate the effect of heat stress on the cecal microbiota, metagenomic sequencing was performed on cecal contents from the CON and HS groups, with four biological replicates per group. As shown in Table 2, the sequencing data were of high quality and suitable for subsequent metagenomic analysis. PCoA revealed a clear separation between the two groups, indicating that heat stress altered the cecal microbial community structure (Figure 5A). In addition, the alpha diversity indices, including ACE, Chao, Simpson, and Shannon, were all significantly higher in the HS group than in the CON group (Figure 5B–E). ANOSIM analysis further confirmed a significant difference in microbial community structure between the two groups (Figure 5F). LEfSe analysis identified distinct differential genera between groups. The CON group was characterized by higher abundances of *Faecalibacterium*, *Colidextribacter*, *Oscillibacter*, *Flavonifractor*, and *Pseudoflavonifractor*, whereas the HS group was characterized by higher abundances of *Mycoplasma*, *Clostridium*, *Thomasclavelia*, *Blautia*, and *Ruminococcus* (Figure 5G).

KEGG functional analysis further revealed significant differences in microbial metabolic pathways between the CON and HS groups. Compared with the CON group, the HS group showed higher relative abundances of pathways related to benzoate degradation, glyoxylate and dicarboxylate metabolism, arabinogalactan biosynthesis—Mycobacterium, O-antigen repeat unit biosynthesis, and biosynthesis of various antibiotics. In contrast, the CON group exhibited higher relative abundances of pathways involved in alanine, aspartate and glutamate metabolism, phenylalanine metabolism, amino sugar and nucleotide sugar metabolism, phosphatidylinositol signaling system, glucosinolate biosynthesis, lysine degradation, quorum sensing, valine, leucine and isoleucine degradation, ABC transporters, two-component system, mannose type O-glycan biosynthesis, and other types of O-glycan biosynthesis (Figure 5H).

### 3.6. Heat Stress Altered the Cecal Metabolomic Profile

**Figure 6:**
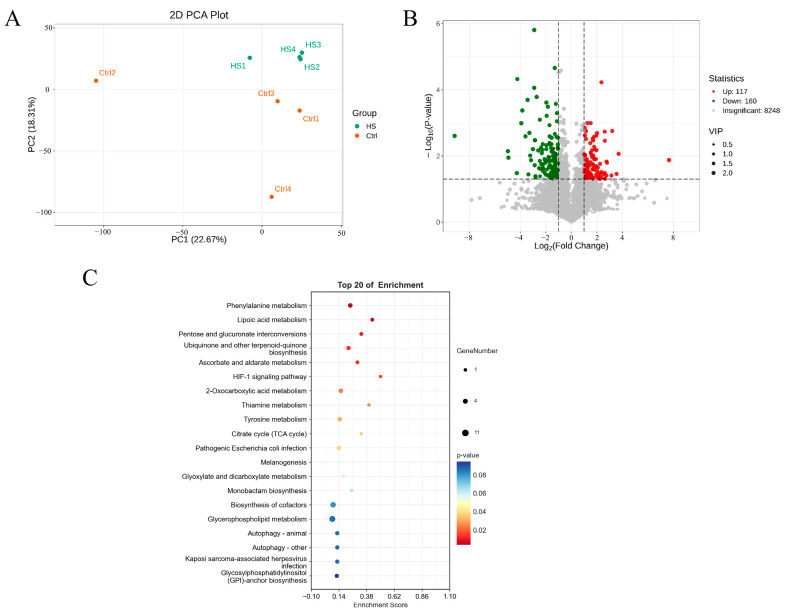
Heat stress altered the cecal metabolomic profile in laying hens. (**A**) PCA score plot of cecal metabolites. (**B**) Volcano plot of differential metabolites between groups. (**C**) Top 20 enriched KEGG pathways of differential metabolites.

To characterize metabolic alterations in the cecal contents under heat stress, metabolomic analysis was performed in the CON and HS groups. PCA revealed a clear separation between the two groups, indicating distinct cecal metabolomic profiles in the CON and HS groups (Figure 6A). Based on the screening criteria, a total of 277 differential metabolites were identified between the two groups, including 117 upregulated and 160 downregulated metabolites in the HS group compared with the CON group (Figure 6B). KEGG enrichment analysis showed that these differential metabolites were mainly enriched in phenylalanine metabolism, lipoic acid metabolism, pentose and glucuronate interconversions, ubiquinone and other terpenoid–quinone biosynthesis, ascorbate and aldarate metabolism, HIF-1 signaling pathway, 2-oxocarboxylic acid metabolism, thiamine metabolism, tyrosine metabolism, citrate cycle (TCA cycle), glyoxylate and dicarboxylate metabolism, glycerophospholipid metabolism, and autophagy-related pathways (Figure 6C).

### 3.7. Correlation Analysis Linked Cecal Microbial and Metabolic Alterations with Host Physiological Changes

**Figure 7:**
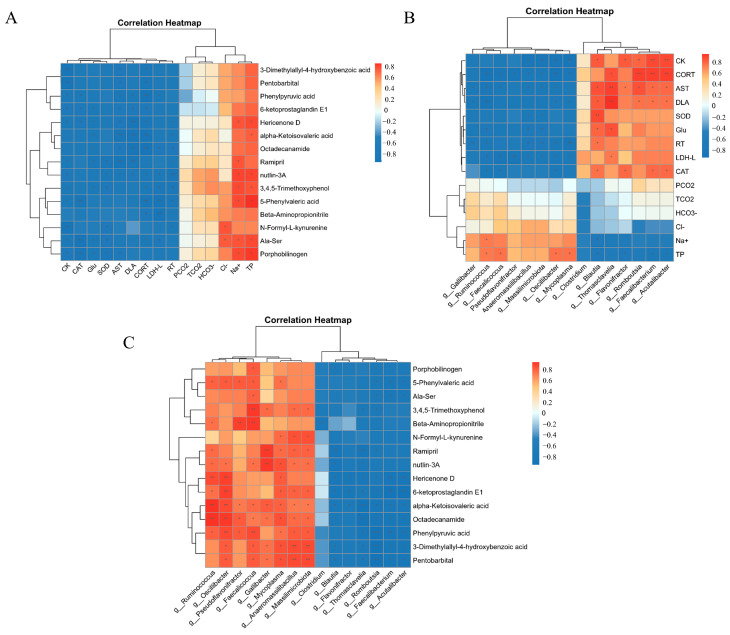
Correlation analysis of cecal microbial, metabolic, and host physiological changes in laying hens under heat stress. (**A**) Correlation heatmap between differential metabolites and host physiological indicators. (**B**) Correlation heatmap between differential metabolites and representative bacterial genera. (**C**) Correlation heatmap between representative bacterial genera and host physiological indicators. Red indicates positive correlations, and blue indicates negative correlations. * *p* < 0.05, ** *p* < 0.01, *** *p* < 0.001.

To further integrate the changes in cecal microbiota, metabolites, and host physiological traits, Spearman correlation analysis was performed. Overall, the selected differential metabolites showed two opposite correlation patterns with host traits. They were generally negatively correlated with heat-stress- and injury-related indicators, including rectal temperature, corticosterone, AST, LDH, CK, D-lactic acid, and Glu, but positively correlated with blood gas, electrolyte, and nutrient-related parameters, such as PCO_2_, TCO_2_, HCO_3_^−^, Cl^−^, Na^+^, and TP (Figure 7A).

A similar pattern was observed at the genus level. The representative genera were separated into two major clusters according to their correlations with host indicators. Genera such as *Clostridium*, *Blautia*, and *Thomasclavelia* were positively correlated with rectal temperature, corticosterone, AST, LDH, CK, D-lactic acid, and Glu, but negatively correlated with PCO_2_, TCO_2_, HCO_3_^−^, Cl^−^, Na^+^, and TP (Figure 7B). In contrast, genera such as *Oscillibacter*, *Pseudoflavonifractor*, and *Ruminococcus* showed the opposite trend. In addition, the selected differential metabolites also displayed consistent positive or negative associations with these two bacterial clusters (Figure 7C). These findings indicate significant associations among cecal microbial alterations, metabolic changes, and heat stress-related physiological disturbances in laying hens. However, the present correlation analyses do not establish causality, and further functional studies, such as fecal microbiota transplantation or gnotobiotic models, are needed to clarify the causal relationships underlying these interactions.

## 4. Discussion

Heat stress is a major challenge to the productivity and health of laying hens because it disrupts physiological homeostasis and metabolic function [1,8,9,27,51]. In the present study, acute heat stress reduced laying rate and eggshell strength, altered serum biochemical, blood gas, and electrolyte parameters, caused liver and intestinal injury, and induced marked changes in hepatic transcriptomic, cecal metagenomic, and cecal metabolomic profiles. These findings indicate that heat stress affects laying hens at multiple biological levels and triggers coordinated host and intestinal responses.

The observed reduction in egg production and eggshell strength suggests that productive performance may be affected under thermal challenge. Notably, egg production was evaluated over the 24 h period following heat stress exposure, indicating a short-term post-stress response rather than an immediate effect during the exposure period. This observation is consistent with previous studies reporting that heat stress can impair egg production and egg quality in laying hens, potentially due to the reallocation of nutrients and energy from production to thermoregulation and maintenance [1,7]. The increase in rectal temperature and corticosterone further confirmed a pronounced stress response. Meanwhile, the increase in blood pH together with the decreases in PCO_2_ and TCO_2_ suggests a tendency toward respiratory alkalosis, likely caused by excessive panting [31]. The concurrent decreases in Na^+^, K^+^, and Cl^−^ indicate that electrolyte homeostasis was also disturbed [52]. Because eggshell formation depends on stable acid–base and mineral balance, these changes may partly explain the reduction in eggshell strength [9,53]. In addition, elevated AST, LDH, CK, and D-lactic acid, together with decreased albumin, triglyceride, cholesterol, and total protein, indicate that heat stress induced broad physiological disturbance involving tissue injury, nutrient imbalance, and altered metabolic status [9,54].

The histological results further showed that the liver and intestine were major targets of heat stress. The liver displayed inflammatory foci, hepatocellular steatosis, cytoplasmic vacuolation, and venous congestion, indicating both inflammatory and degenerative injury, which is consistent with recent reports describing hepatic lipid metabolic disorder, oxidative imbalance, and inflammatory damage in heat-stressed poultry [55]. At the intestinal level, shortened villi, increased crypt depth, and reduced villus height/crypt depth ratio, particularly in the ileum and duodenum, suggest impaired absorptive capacity and accelerated epithelial turnover, in agreement with recent studies showing that heat stress compromises intestinal morphology and epithelial barrier integrity in poultry [56]. Such structural damage is biologically important because intestinal injury may reduce nutrient utilization and increase host exposure to luminal microbial products, thereby aggravating gut dysfunction under heat stress [57]. Therefore, the combined liver and intestinal damage observed here likely contributed to the systemic metabolic disturbance induced by heat stress.

The hepatic transcriptomic data provide mechanistic support for these pathological changes. Heat stress activated inflammatory and stress-responsive pathways, including Toll-like receptor, NOD-like receptor, MAPK, cytokine-related, apoptosis, and ECM–receptor interaction pathways, indicating enhanced innate immune signaling, tissue injury responses, and extracellular remodeling in the liver, which is consistent with recent transcriptomic evidence showing that heat stress rapidly triggers signaling adjustment and immune- and stress-related responses in chicken liver [58]. The agreement between these molecular changes and the histological evidence of hepatic inflammation, together with the increases in serum injury markers, further supports the interpretation that the liver is a major target of heat stress-induced injury in poultry [59]. At the same time, heat stress inhibited pathways related to lipid utilization, antioxidant defense, and energy production, including PPAR signaling, glutathione metabolism, fatty acid degradation, the citrate cycle, and oxidative phosphorylation, in line with recent omics studies showing that heat stress alters hepatic metabolism, oxidative phosphorylation, lipid metabolism, and immune responses in poultry tissues [60]. This pattern suggests that the liver shifted from a metabolically active state toward an inflammatory and injury-responsive state under heat stress. Moreover, suppression of fatty acid degradation, antioxidant metabolism, and mitochondrial energy pathways may help explain the hepatic steatosis and reduced metabolic efficiency observed under thermal challenge [10]. Thus, the hepatic response to heat stress was characterized by the coexistence of inflammatory activation and metabolic suppression.

Heat stress also markedly reshaped the cecal microbial ecosystem. The clear separation between groups in community structure analysis indicates that the cecal microbiota was substantially altered by thermal challenge, which is consistent with recent evidence showing that heat stress induces taxonomic shifts in the chicken gut microbiome and can produce both acute and longer-term changes in cecal microbial structure [61]. The higher abundances of *Clostridium*, *Thomasclavelia*, and *Blautia* in the HS group, together with the enrichment of *Faecalibacterium*, *Oscillibacter*, *Flavonifractor*, and *Pseudoflavonifractor* in the CON group, suggest that heat stress changed the ecological balance of the cecal community. Although genus-level interpretations should be made cautiously, this overall shift agrees with previous reports that heat stress alters intestinal microbial composition and gut health in poultry [62]. Importantly, microbial functional analysis further showed that heat stress increased benzoate degradation and glyoxylate and dicarboxylate metabolism, while reducing pathways related to phenylalanine metabolism, alanine, aspartate and glutamate metabolism, ABC transporters, and the two-component system. These results indicate that heat stress affected not only microbial composition but also microbial metabolic and regulatory potential.

Acute heat stress significantly increased cecal alpha diversity in the present study, indicating that short-term thermal challenge rapidly altered the intestinal microbial ecosystem. Previous studies investigating the effects of heat stress on poultry gut microbiota have reported inconsistent findings regarding alpha diversity, with some studies showing no significant changes or even decreases, whereas others observed increased richness and evenness indices [57,63,64]. Recent reviews have suggested that these discrepancies may be associated with differences in the duration and intensity of heat stress, animal age, dietary background, and the initial microbial composition prior to heat exposure. Compared with chronic heat stress models, acute heat stress may induce rapid physiological and intestinal environmental disturbances without causing a complete collapse of microbial community structure. In our study, acute heat stress markedly increased serum DAO and D-LA levels, indicating impaired intestinal barrier integrity and increased intestinal permeability. Damage to the intestinal epithelium may transiently weaken its protective function and alter nutrient availability and oxygen gradients within the intestinal lumen, thereby creating temporary ecological niches that facilitate microbial redistribution and the opportunistic expansion of diverse bacterial taxa. Previous studies have suggested that heat stress may suppress beneficial bacterial populations while promoting the proliferation of opportunistic or potentially pathogenic microorganisms, which could contribute to elevated alpha diversity indices [65,66,67]. Therefore, the increased alpha diversity observed in this study likely reflects a rapid microbial adaptive response to acute intestinal environmental disturbance induced by heat stress rather than a beneficial enhancement of microbial stability [15]. These findings further suggest that acute heat stress can rapidly reshape the intestinal microbial ecosystem, even within a relatively short exposure period.

The cecal metabolomic results further supported the presence of broad metabolic disturbance under heat stress. Differential metabolites were mainly enriched in phenylalanine metabolism, lipoic acid metabolism, glyoxylate and dicarboxylate metabolism, the citrate cycle, and glycerophospholipid metabolism, indicating alterations in amino acid, redox, energy, and lipid-related metabolism. This interpretation is consistent with recent studies showing that heat stress reshapes metabolic profiles in poultry and is frequently associated with disrupted amino acid, lipid, and energy metabolism [64]. Notably, several of these pathways overlapped with those identified in the metagenomic functional analysis and hepatic transcriptomic data, particularly glyoxylate and dicarboxylate metabolism, phenylalanine-related metabolism, and the citrate cycle. Similar cross-omics patterns have recently been reported in poultry, where heat stress was shown to induce coordinated transcriptomic and metabolomic changes and to alter gut microbe–metabolite interactions associated with host physiological regulation [68,69]. This cross-omics consistency suggests that heat stress induced coordinated metabolic reprogramming involving both the host and the intestinal environment.

The integrated analysis of hepatic transcriptomics, cecal metagenomics, and cecal metabolomics indicates that acute heat stress induced coordinated inflammatory activation and metabolic disturbance in laying hens, which is consistent with recent multi-omics evidence showing that heat stress can simultaneously alter host gene expression, intestinal microbiota, and metabolic profiles in poultry [70]. In the present study, hepatic transcriptomics revealed inflammatory activation and metabolic suppression, whereas cecal metagenomics and metabolomics showed concurrent remodeling of microbial structure, microbial function, and intestinal metabolic output [71]. The correlation analysis further revealed coordinated association patterns among host physiological indicators, microbial genera, and differential metabolites under acute heat stress. Stress- and injury-related host indicators exhibited clustering patterns opposite to those of blood gas, electrolyte, and nutrient-related parameters, while representative bacterial genera and differential metabolites displayed corresponding associations. These findings are consistent with previous studies showing close associations between host physiological traits and transcriptomic or metabolomic signatures under heat stress conditions in laying hens [72]. However, because these analyses are correlative in nature, causal relationships among microbial alterations, metabolic changes, and host physiological responses cannot be established in the present study. Further functional studies, such as fecal microbiota transplantation or gnotobiotic models, are needed to clarify the mechanistic interactions underlying these associations.

Taken together, this study provides an integrated multi-omics framework linking acute heat stress to coordinated alterations in hepatic transcription, cecal microbiota, and metabolic outputs along the gut–liver axis. Importantly, the observed changes in serum biochemical indicators, including D-lactic acid and aspartate aminotransferase (AST), suggest their potential utility as early biomarkers for heat-stress-induced physiological disturbances in laying hens. However, the interpretation of these findings should be considered within the limitations of the current experimental design, which also highlights important directions for future research. Although the consistency across transcriptomic, metagenomic, and metabolomic datasets supports the robustness of the observed patterns, the moderate sample size for omics analyses may limit the resolution of inter-individual variability, and larger independent cohorts are required for validation. Feed intake was not individually monitored during the acute heat stress period; therefore, its potential contribution to the observed physiological and multi-omics changes cannot be excluded, and future studies incorporating pair-fed or intake-controlled designs are warranted. In addition, the acute heat stress model reflects short-term responses rather than long-term adaptation, and longitudinal studies are needed to elucidate the temporal dynamics of the gut–liver axis. Finally, the observed associations among host physiology, hepatic gene expression, microbiota, and metabolites are correlative, and causal relationships remain to be established using functional approaches such as fecal microbiota transplantation or gnotobiotic models. Collectively, these limitations should be considered when interpreting the present findings and will guide future mechanistic and controlled investigations of heat stress responses in laying hens.

## 5. Conclusions

In conclusion, this study demonstrates that acute heat stress rapidly impairs the productive performance and tissue homeostasis of laying hens through a coordinated mechanism involving the gut–liver axis. Our integrated multi-omics analysis specifically reveals that heat-induced injury is driven by profound hepatic metabolic suppression and concurrent inflammatory activation. Additionally, we identified significant shifts in cecal microbial communities and energy-related metabolites. Together, these findings elucidate the critical microbiota–host metabolic crosstalk during early thermal challenge, providing potential targets, such as modulation of the gut microbiota or mitigation of hepatic inflammation, for future nutritional interventions in the poultry industry.

## Figures and Tables

**Table 1:** Clean sequencing data of liver samples after quality control.

Sample	Raw_Reads	Raw_Bases	Clean_Reads	Clean_Bases	Q20	Q30	Unique_Map
CON1	44,375,236	6.66 G	43,437,002	6.52 G	96.71	91.56	90.12%
CON2	40,208,518	6.03 G	39,251,040	5.89 G	96.83	91.79	91.15%
CON3	45,271,324	6.79 G	44,046,088	6.61 G	96.93	92.00	91.29%
CON4	42,378,030	6.36 G	41,499,178	6.22 G	97.03	92.15	91.57%
HS1	42,464,888	6.37 G	41,669,998	6.25 G	97.19	92.43	92.34%
HS2	42,707,702	6.41 G	41,719,082	6.26 G	96.72	91.56	90.92%
HS3	40,537,004	6.08 G	39,069,864	5.86 G	96.72	91.53	90.59%
HS4	42,913,474	6.44 G	42,088,718	6.31 G	96.98	92.07	91.43%

**Table 2:** The summary of the Metagenomics sequencing data.

Sample	Raw_Bases	Clean_Bases	Clean_Q20	Clean_Q30	Clean_GC	Effective
CON1	12.29 G	12.01 G	99.50%	98.10%	53.69%	97.74%
CON2	13.12 G	12.93 G	99.44%	97.88%	54.17%	98.57%
CON3	10.03 G	9.95 G	99.41%	97.76%	56.34%	99.23%
CON4	10.00 G	9.93 G	99.42%	97.84%	58.78%	99.25%
HS1	18.31 G	18.29 G	99.39%	97.94%	49.23%	99.88%
HS2	20.80 G	20.58 G	99.49%	98.33%	50.38%	98.93%
HS3	16.88 G	16.87 G	99.42%	98.01%	49.73%	99.91%
HS4	20.56 G	20.51 G	99.41%	98.06%	49.24%	99.75%

## Data Availability

The datasets presented in this study are available in the NCBI Sequence Read Archive (SRA) under BioProject accession number PRJNA1456219.

## Abbreviations

The following abbreviations are used in this manuscript:

ALB	Albumin
AST	Aspartate aminotransferase
CAT	Catalase
CK	Creatine kinase
CORT	Corticosterone
DAO	Diamine oxidase
DLA	D-lactic acid
GLU	Glucose
GO	Gene Ontology
GSEA	Gene set enrichment analysis
Hb	Hemoglobin
HS	Heat stress group
KEGG	Kyoto Encyclopedia of Genes and Genomes
LDH	Lactate dehydrogenase
LEfSe	Linear discriminant analysis effect size
MAPK	Mitogen-activated protein kinase
PCA	Principal component analysis
PCoA	Principal coordinate analysis
PCO_2_	Partial pressure of carbon dioxide
PPAR	Peroxisome proliferator-activated receptor
qRT-PCR	Quantitative real-time polymerase chain reaction
RNA-seq	RNA sequencing
SOD	Superoxide dismutase
TCHO	Total cholesterol
TCO_2_	Total carbon dioxide
TG	Triglyceride
TP	Total protein

## Appendix A

**Table A1:** Primers used for qPCR.

Primer Name	Primer Sequence (5′-3′)	TM (°C)	Product Length (bp)
TLR4-F	TGGATCTTTCAAGGTGCCACA	59.86	198
TLR4-R	AGTGTCCGATGGGTAGGTCA	59.96	
PTGS2-F	GACTCCGAAGTCAGTGCAGC	61.01	294
PTGS2-R	CACGTGAAGAATTCCGGTGTTG	60.35	
PPARG-F	GATTTCTCAACCGCAGCGAG	59.63	179
PPARG-R	ACACCACAGGAACTTGCACT	59.74	
BCL2L1-F	TGACCACGTACTTGACCGAC	59.69	72
BCL2L1-R	GATCCACAAAGCGCTCCCAG	61.37	
CYP1A1-F	CAGCACCCAGAGGTTCACTT	59.89	293
CYP1A1-R	CCGATGGTCACCTCCATCAC	60.18	
β-actin-F	CAGCCAGCCATGGATGATGA	60.18	150
β-actin-R	CATACCAACCATCACACCCTGA	60.03	
